# Human epididymis protein 4 aggravates airway inflammation and remodeling in chronic obstructive pulmonary disease

**DOI:** 10.1186/s12931-022-02040-7

**Published:** 2022-05-12

**Authors:** Yuan Zhan, Jinkun Chen, Jixing Wu, Yiya Gu, Qian Huang, Zhesong Deng, Shanshan Chen, Xiaojie Wu, Yongman Lv, Zhilin Zeng, Jungang Xie

**Affiliations:** 1 Department of Respiratory and Critical Care Medicine, National Clinical Research Center of Respiratory Disease, Key Laboratory of Pulmonary Diseases of Health Ministry, Tongji Hospital, Tongji Medical College, Huazhong University of Science and Technology, 1095 Jiefang Road, Wuhan, 430030 Hubei China; 2 Department of Science, Western University, 1151 Richmond Street, London, ON N6A 3K7 Canada; 3 Department of Respiratory and Critical Care Medicine, Wuhan NO.1 Hospital, Wuhan Hospital of Traditional Chinese and Western Medicine, Wuhan, 430022 China; 4 Health Management Center, Tongji Hospital, Tongji Medical College, Huazhong University of Science and Technology, Wuhan, 430030 Hubei China; 5 Department and Institute of Infectious Diseases, Tongji Hospital, Tongji Medical College, Huazhong University of Science and Technology, 1095 Jiefang Road, Wuhan, China

**Keywords:** Human epididymis protein 4, Airway epithelium inflammation, Fibroblast differentiation and proliferation, Chronic obstructive pulmonary disease

## Abstract

**Background:**

Chronic obstructive pulmonary disease (COPD) is a progressive disease characterized by chronic inflammation and airway remodeling. Human epididymis protein 4 (HE4) plays a critical role in various inflammatory or fibrotic diseases. However, the role of HE4 in COPD remains unidentified.

**Methods:**

HE4 expression was determined in the lung tissues from COPD patients and cigarette smoke (CS)-exposed mice using immunohistochemical staining, qPCR, or western blot. The plasma level of HE4 was detected by ELISA. The regulations of HE4 in the expressions of CS extract (CSE)-induced inflammatory cytokines in human bronchial epithelial cells (HBE) were investigated through knockdown or overexpression of HE4. The role of secretory HE4 (sHE4) in the differentiation and proliferation in human pulmonary fibroblast cells (HPF) was explored via qPCR, western blot, CCK8 assay or 5-ethynyl-2′-deoxyuridine (EdU) staining. The probe of related mechanism in CSE-induced HE4 increase in HBE was conducted by administrating N-acetylcysteine (NAC).

**Results:**

HE4 was up-regulated in both the lung tissue and plasma of COPD patients relative to controls, and the plasma HE4 was negatively associated with lung function in COPD patients. The same enhanced HE4 expression was verified in CS-exposed mice and CSE-induced HBE, but CSE failed to increase HE4 expression in HPF. In vitro experiments showed that reducing HE4 expression in HBE alleviated CSE-induced IL-6 release while overexpressing HE4 facilitated IL-6 expression, mechanistically through affecting phosphorylation of NFκB-p65, whereas intervening HE4 expression had no distinctive influence on IL-8 secretion. Furthermore, we confirmed that sHE4 promoted fibroblast-myofibroblast transition, as indicated by promoting the expression of fibronectin, collagen I and α-SMA via phosphorylation of Smad2. EdU staining and CCK-8 assay demonstrated the pro-proliferative role of sHE4 in HPF, which was further confirmed by enhanced expression of survivin and PCNA. Pretreatment of NAC in CSE or H_2_O_2_-induced HBE mitigated HE4 expression.

**Conclusions:**

Our study indicates that HE4 may participate in airway inflammation and remodeling of COPD. Cigarette smoke enhances HE4 expression and secretion in bronchial epithelium mediated by oxidative stress. Increased HE4 promotes IL-6 release in HBE via phosphorylation of NFκB-p65, and sHE4 promotes fibroblastic differentiation and proliferation.

**Supplementary Information:**

The online version contains supplementary material available at 10.1186/s12931-022-02040-7.

## Background

Chronic obstructive pulmonary disease (COPD) is the most prevalent respiratory disease of great threaten to health, and meanwhile is the third leading cause of death worldwide with substantial socioeconomic and medical burden [1]. As a progressive disease associated with destruction of the lung parenchyma and small airway lesion, COPD is clinically characterized by persistent airflow limitation and impaired pulmonary function [2]. Tobacco smoking remains the major risk factor accounting for COPD morbidity and death [3], and multiple studies established the associations between other environmental contributors and COPD pathogenesis, such as indoor air pollution from solid fuels and ambient fine particulate matters [4, 5]. Moreover, several processes have been indicated to potentially involved in the pathogenesis of COPD, including the overexpression of inflammatory mediators and cytokines, the activation of inflammatory signaling pathways, the protease/anti-protease imbalance, and the oxidation–antioxidation imbalance [6]. However, as of now, there is limited detailed insight into the cellular and molecular mechanism underlying the pathophysiology of COPD.

Oxidative stress is conceived as a critical mechanism for COPD pathogenesis. COPD patients suffer from oxidative stress caused by the inhalation of cigarette smoke (CS), which leads to excessive production of reactive oxygen species (ROS) [7], and thereafter results in airway epithelium injury and accumulation of various inflammatory mediators, aggravating the development of COPD [8, 9]. Meanwhile, an increasing body of evidence indicates that airway remodeling, another hallmark feature of COPD pathological change, is subjected to multiple events including epithelial abnormalities [10], fibroblast differentiation and proliferation [11], airway smooth muscle hypertrophy and hyperplasia [12]. The bronchial epithelium cells, as the first defensive barrier in response to CS, can trigger immune and inflammatory processes by secreting various mediators such as cytokines, chemokines, growth factors, lipid mediators and other bioactive particles [13, 14]. In addition, some secretory factors released from the epithelial cells, like TGF-β and IL-6, can impact the function of fibroblast containing differentiation to myofibroblast, proliferation and extracellular matrix deposition [15, 16]. Taken together, bronchial epithelial cell injury and fibroblast function changes play pivotal roles in promoting the development of COPD, and some cross-talk may exist between these two types of cells. But the concrete mechanism there into needs to be confirmed by more researches.

Human epididymis protein 4 (HE4), also known as WAP four-disulfide core domain protein 2, has been investigated in various tumorous and fibrotic diseases [17–20]. Also, recent studies have revealed that HE4 served as a crucial molecule/factor in immune-modulation and host defense [21, 22]. Lin and his colleagues have shown that serum HE4 was associated with cardiovascular events of COPD patients [23]. However, the role and related action mechanism of HE4 in COPD remain obscure.

In the present study, we aimed to explore the role of HE4 in airway inflammation and remodeling for COPD. Specifically, we successively measured the expression levels of HE4 in the lung tissue and peripheral blood plasma of COPD patients. The association between plasma HE4 level and lung function was evaluated. Moreover, the changes in HE4 expression was further confirmed in CS-exposed mice and CS extract (CSE)-treated bronchial epithelial cells (HBE). In in vitro experiments, we investigated the effects of HE4 on inflammatory cytokines and related regulation mechanisms in CSE-exposed HBE, and probed whether the secretory HE4 protein from HBE impacted fibroblast differentiation and proliferation, participating in airway remodeling. We believe the findings above will be of great value to further elucidation for COPD pathogenesis.

## Materials and methods

### Clinical subjects

All subjects including peripheral blood plasma and lung tissues in this study were recruited from Tongji Hospital, Wuhan, China, between 2019 and 2021. Thereinto, the lung specimens were collected from patients who underwent surgical resection for pulmonary lump in the department of thoracic surgery. All participants were stratified into three groups based on the smoking history and lung function, namely non-smokers, smokers and COPD. COPD was diagnosed according to the Global Initiative for Chronic Obstructive Lung Disease (GOLD) 2020 criteria. Patients with a post-bronchodilator forced expiratory volume in 1 s (FEV_1_)/forced vital capacity ratio of less than 70% were enrolled. Age- and gender-matched non-smokers and smokers without COPD were also recruited as control subjects. Patients were excluded if they suffered from asthma, severe lung infections, or other obstructive lung diseases. This study was approved by the Ethics Committees of the Tongji Hospital (TJ-IRB20210346) and written informed consent was obtained from all subjects.

### Sample collection and deposal

The lung tissues were collected at least 5 cm away from the lesion and fixed with formalin, then embedded in paraffin, or were homogenized and stored at − 80 °C. Plasma was separated from blood cells by centrifugation at 3000 rounds per minute (rpm) for 8 min and stored at − 80 °C until detection.

### Preparation of cigarette smoke extract

Cigarette smoke extract (CSE) was prepared by bubbling the smoke from two burning cigarettes (3R4F, University of Kentucky) at a rate of 1 cigarette/5 min to a 50 ml centrifuge tube containing 20 ml of RPMI-1640 medium. The pH was adjusted to 7.4. The solution was then filtered through a 0.22 μm filter to eliminate bacteria.

### Cell culture and treatment

Human lung bronchial epithelial cell line (HBE) was obtained from the Boster Biotech Co., Ltd (Wuhan, China). Cells were cultured in RPMI 1640 medium supplemented with 10% fetal bovine serum and 1% penicillin/streptomycin in a humidified incubator under 5% CO_2_ at 37 °C. Human pulmonary fibroblast (HPF) was purchased from ScienCell Research Laboratories, Inc (Nanjing, China). Cells were maintained in fibroblast medium supplemented with 10% fetal bovine serum and 1% penicillin/streptomycin in the same environment as HBE culture.

HBEs were treated with CSE at different concentrations for 24 h. In experiments of HE4 knockdown, HBEs were transfected with 50 nM small interfering RNA (siRNA) targeting HE4 (5′-CCCAGGTGAACATTAACTT-3′) or negative control sequence (RiboBio, Guangzhou, China) using Lipofectamine 3000 (Invitrogen, Carlsbad, CA, United States) according to the manufacturer’s instructions. In experiments of HE4 overexpression, HBEs were infected with lentiviral vector (Negative Control or HE4-overexpressing) purchased from the OBiO Biotechnology (Shanghai, China) following the manufacturer’s instructions. The efficiency of knockdown or overexpression was determined using real-time qPCR and Western blot assay. In pharmacological experiment, HBEs were pretreated with 3 mM N-Acetylcysteine (Sigma-Aldrich, St. Louis, MO, United States) for 1 h before the 24-h stimulation of CSE or H_2_O_2_. And various concentrations of Bay 11-7821 (MedChemExpress, United States) were delivered to HBEs. Moreover, HPFs were treated by recombinant human HE4 protein (rHE4) (Absin, Shanghai, China) at different concentrations.

### Animal sample

All animal samples including mouse lung tissue sections, RNA and protein were obtained from our research team. The animal models were introduced as described previously [24]. Briefly, Male C57BL/6 J wild-type mice, 10–12 weeks old, were exposed to cigarette smoke in a chamber using a modified method of 10 cigarettes for two separate 2-h periods per day, 5 days per week continuously for 6 months. Thereinto, the mice in cigarette smoke-exposed group and air control group were detected for HE4 expression. Animal experiments were approved by the ethics committee of Tongji Hospital, Huazhong University of Science and Technology.

### Immunohistochemical analysis

Immunohistochemical assay was performed with HE4 antibody (1:200, ABclonal Technology, Wuhan, China) in human and mouse lung tissue sections. Formalin (10%)-fixed, paraffin-embedded lung tissue sections were deparaffinized using xylene and rehydrated in a graded ethanol series. Heat-induced antigen retrieval was performed using a microwave. After cooling with running tap water, sections were incubated with 3% hydrogen peroxide to block endogenous peroxidase activity, followed by 1-h incubation in 5% BSA-phosphate-buffered saline solution at room temperature to avoid non-specific background. Then, slides were incubated with HE4 antibody at 4 °C overnight in a humidified chamber. After washing, sections were incubated with a peroxidase-conjugated goat anti-rabbit secondary antibody for 1 h at room temperature. The reactions were developed using a DAB substrate kit with hematoxylin as a counterstain. A Nikon Spot image system (United States) was used for image acquisition.

### Real-time qPCR

Total RNA was extracted using Trizol (Takara, Japan) and reversely transcribed to cDNA using the cDNA RT-PCR Kit (Takara, Japan). The mRNA expression was assessed by real-time quantitative polymerase chain reaction (RT-qPCR) using TB GreenR Premix Ex TaqTM II (Takara, Japan) on BioRad CFX384 (Bio-Rad, CA, United States). The parameters were as follows: 40 cycles at 95 °C for 10 s, 59 °C for 20 s, and 72 °C for 30 s. Data were analyzed using the 2^−ΔΔCt^ method with β-actin as control. The detailed information of all primers was shown in Table 1.

**Table 1:** Primer information

Genes	Primer pairs (5′–3′)
Human HE4	AGAACTGCACGCAAGAGTG
	TTGAGGTTGTCGGCGCATT
Human β-actin	AGAAAATCTGGCACCACACCT
	GATAGCACAGCCTGGATAGCA
Human IL-6	CTGCTGCCTTCCCTGCC
	CCTCTTTGCTGCTTTCACACAT
Human IL-8	AAGAAACCACCGGAAGGAAC
	ACTCCTTGGCAAAACTGCAC
Human Fibronectin	GAGAATAAGCTGTACCATCGCAA
	CGACCACATAGGAAGTCCCAG
Human Collagen I	GAGGGCCAAGACGAAGACATC
	CAGATCACGTCATCGCACAAC
Human α-SMA	GTGTTGCCCCTGAAGAGCAT
	GCTGGGACATTGAAAGTCTCA
Mouse he4	GTTGCTACTGTTCACCCCCA
	GGTGCCCTGCTTTTCATTAGG
Mouse β-actin	AGAAAATCTGGCACCACACCT
	GATAGCACAGCCTGGATAGCA

### Western blot

Total proteins were prepared using RIPA lysis buffer containing phosphatase inhibitors. The concentration was determined by BCA assay (Bioyear Biotechnology, Wuhan, China). Equal amounts of protein were subjected to 10% SDS-PAGE and transferred to polyvinylidene fluoride (PVDF) membranes. The membranes were blocked with 5% non-fat milk and incubated with the appropriate primary antibodies: anti-HE4 (1:1000, ABclonal Technology, Wuhan, China), anti-β-actin (1:4000, Proteintech, Wuhan, China), anti-p-p65 (1:1000, Cell Signal Technology, MA, USA), anti-p65 (1:4000, Cell Signal Technology, MA, USA), anti-Flag (1:4000, Sigma-Aldrich, St. Louis, MO, United States), anti-Fibronectin (1:1000, Proteintech, Wuhan, China), anti-collagen I (1:1000, Proteintech, Wuhan, China), anti-α-SMA (1:1000, Cell Signal Technology, MA, USA), anti-p-Smad2 (1:1000, Boster Biotech, Wuhan, China), anti-Smad2 (1:2000, Cell Signal Technology, MA, USA), anti-Survivin (1:2000, Proteintech, Wuhan, China), anti-PCNA (1:4000, Proteintech, Wuhan, China), anti-GAPDH (1:4000, Proteintech, Wuhan, China). After washing with Tris-buffered saline (TBS) containing 0.1% Tween-20, blots were incubated with secondary antibody conjugated to horseradish peroxidase (HRP; 1:4000, Proteintech, Hubei, People’s Republic of China). The intensity of individual bands was quantified using ImageJ.

### Enzyme-linked immunosorbent assay (ELISA)

Concentration of secretory HE4 (sHE4) in human peripheral blood plasma and then the concentrations of sHE4, interleukin-6 (IL-6) and interleukin-8 (IL-8) in cell-culture supernatants were determined using Human HE4, IL-6 and IL-8 DuoSet ELISA kits (R&D Systems, Minneapolis, MN, United States) according to the manufacturer’s instructions. The minimum doses of detection for the HE4, IL-6 and IL-8 ELISA kits were 62.5 pg/mL, 9.38 pg/mL and 31.3 pg/mL, respectively.

### Proliferation detection

Proliferation was assessed by CCK8 assay and 5-ethynyl-2′-deoxyuridine (EdU) staining according to the manufacturer’s instructions. Briefly, cells were seeded in a 96-well plate (Corning, MA, USA) and treated with different concentrations of rHE4 for different time. After treatment, 5 mg/mL CCK 8 solution was added, and cells were incubated at 37 °C for 2 h according to the manufacturer’s instructions (Dojindo Laboratories, Tokyo, Japan). Optical density (OD) values were measured at the wavelength of 450 nm using the Microplate Reader (Bio-Tek Instruments, VT, USA). For EdU staining, the cells were washed with PBS and then incubated with culture medium containing 50 μM EdU (RiboBio, Wuhan, China) for 2 h. The cells were fixed and then subjected to Apollo staining and DNA staining. Images were acquired with a fluorescence microscope (Olympus, Shinjuku, Japan).

### Statistical analysis

All data were expressed as mean ± SEM unless otherwise specified. Statistical significance was determined using Student’s t-test for two groups, or one-way ANOVA with Newman–Keuls post hoc test for multiple comparisons. The correlations were analyzed by Pearson correlation. The GraphPad Prism 8 Software (GraphPad Software, San Diego, CA, United States) was used for all statistical analysis and graphic generation. P-value < 0.05 was considered to be statistically significant.

## Results

### Subject characteristics

The clinical characteristics of all subjects were displayed in Table 2. In all, 78 subjects were included in the current study containing 12 non-smokers, 15 smokers without COPD and 51 smokers with COPD. There presents no obvious differences by age, sex and body mass index (BMI). The smoking history was similar in the patients of smokers without COPD and with COPD. The patients in COPD group had significantly lower FEV_1_% of predicted and FEV_1_/FVC, compared with those in non-smoker group and smoker group. Out of the 78 patients, there were 10, 11, and 11 patients in non-smoker, smoker and COPD groups respectively, who underwent the surgical resection for pulmonary lump, and we collected both the lung tissue and peripheral blood from them. Whereas only the peripheral blood was acquired in the remaining 46 patients.

**Table 2:** Clinical characteristics of subjects

	Non-smokers (n = 12)	Smokers (n = 15)	COPD (n = 51)
Age (years)	59.00 (53.00–67.50)	61.00 (58.00–67.00)	66.00 (57.00–69.00)
Men, n (%)	11 (91.67)	14 (93.33)	50 (98.04)
BMI (kg/m^2^)	23.02 (21.09–24.69)	23.00 (20.69–24.80)	22.36 (19.38–23.95)
Smoking (pack-years)	0	39.00 (32.00–47.00)*	45.00 (39.00–60.00)*
FEV1 (% predicted)	93.00 (89.25–109.50)	88.00 (84.00–93.00)	44.00 (37.00–54.00)*^#^
FEV1/FVC (%)	80.75 (77.11–82.30)	74.30 (72.40–78.25)	47.04 (38.84–60.00)*^#^

Data are expressed as median (IQR) or n (%), *P < 0.05 vs Non-smokers, ^#^P < 0.05 vs Smokers

### HE4 expression was upregulated in the lung and peripheral blood plasma of COPD

**Fig. 1:**
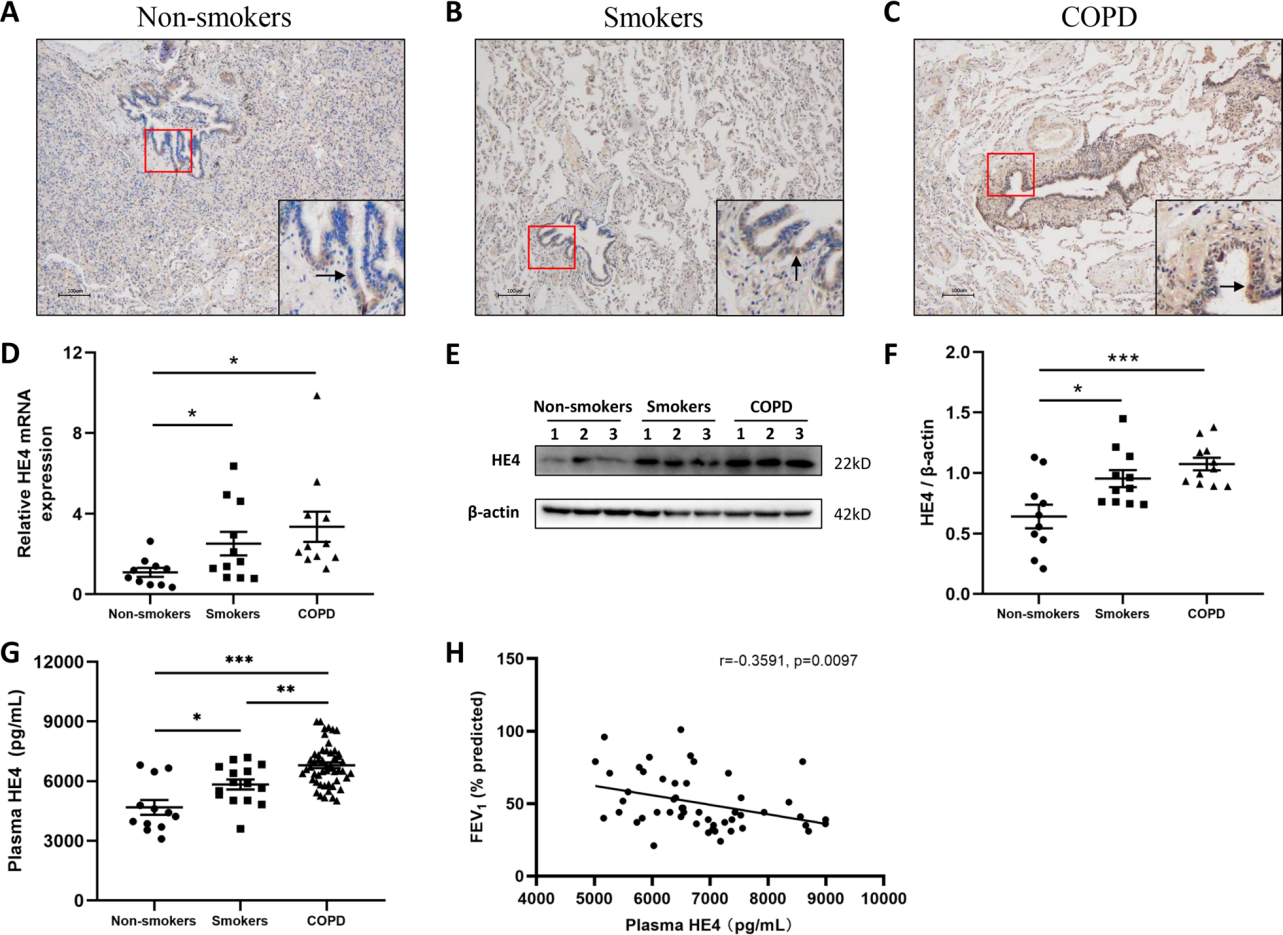
HE4 expression was up-regulated in the lung tissue and peripheral blood plasma of COPD patients. **A–C** Representative immunohistochemical images of human lung tissues against HE4 are shown. The tan staining was mainly presented in the bronchial epithelium. Compared with non-smokers and smokers, the degree and extent of tan staining was more pronounced and extensive in the bronchial epithelium of COPD patients. The staining alteration can be clearly observed by the arrowheads. Magnification = × 100. The red-boxed area indicates an area of higher magnification. **D** HE4 mRNA expression was increased in COPD patients. **E, F** Representative HE4 expression in the lung tissue homogenates was detected by Western blot and quantified using ImageJ. Each lane represents a different lung tissue subject. **G** Increased secretory HE4 was determined in the peripheral blood plasma of COPD patients relative to non-smokers and smokers. **H** Elevated sHE4 expression in plasma of COPD patients was negatively related with lung function. Data are expressed as mean ± SEM. For detection for lung tissues, n = 10 for non-smokers, n = 11 for smokers, and n = 11 for COPD. For detection for peripheral blood plasma, n = 12 for non-smokers, n = 15 for smokers, and n = 51 for COPD. P-values were calculated using one-way ANOVA followed by Newman–Keuls test. *P < 0.05, **P < 0.01, and ***P < 0.001 represent significant differences. HE4, human epididymis protein 4; COPD, chronic obstructive pulmonary disease; sHE4, secretory human epididymis protein 4; FEV_1_%pred, forced expiratory volume in 1 s (FEV_1_) % predicted

To explore the expression of HE4 in COPD patients, we successively examined it in the lung tissues using immunohistochemical staining, PCR and western blot, and then in the peripheral blood plasma using ELISA. As shown in Fig. 1A–C, HE4 expression was mainly located in the bronchial epithelium, and relative to non-smokers and smokers, the staining intensity of tan color was markedly stronger in COPD patients. Thereinto, the tan staining was more pronounced in smokers compared to non-smokers. Then, we revealed that the intrapulmonary HE4 expression was successively up-regulated in non-smokers, smokers and COPD patients both at mRNA and protein levels (Fig. 1D–F), despite that there presented no statistically difference in smoker group and COPD group, which was potentially ascribed to the limited sample size.

It was reported that HE4 could be used as possible biomarker signifying the extent of disease progress [25, 26]. Therefore, we measured the HE4 level in the peripheral blood plasma via ELISA, and found the highest HE4 content in COPD patients, followed by the smokers and non-smokers (Fig. 1G). Nagy and colleagues have reported HE4 as a novel serum inflammatory biomarker in cystic fibrosis [21]. In our study, we discovered that in COPD patients, the expression level of plasma HE4 was negatively associated with FEV_1_% predicted (Fig. 1H), which indicated the potential impact of HE4 on the declined lung function and COPD process. The plasma HE4 may serve as a biomarker indicating disease severity of COPD patients.

### He4 expression was increased in cigarette smoke-exposed mice

**Fig. 2:**
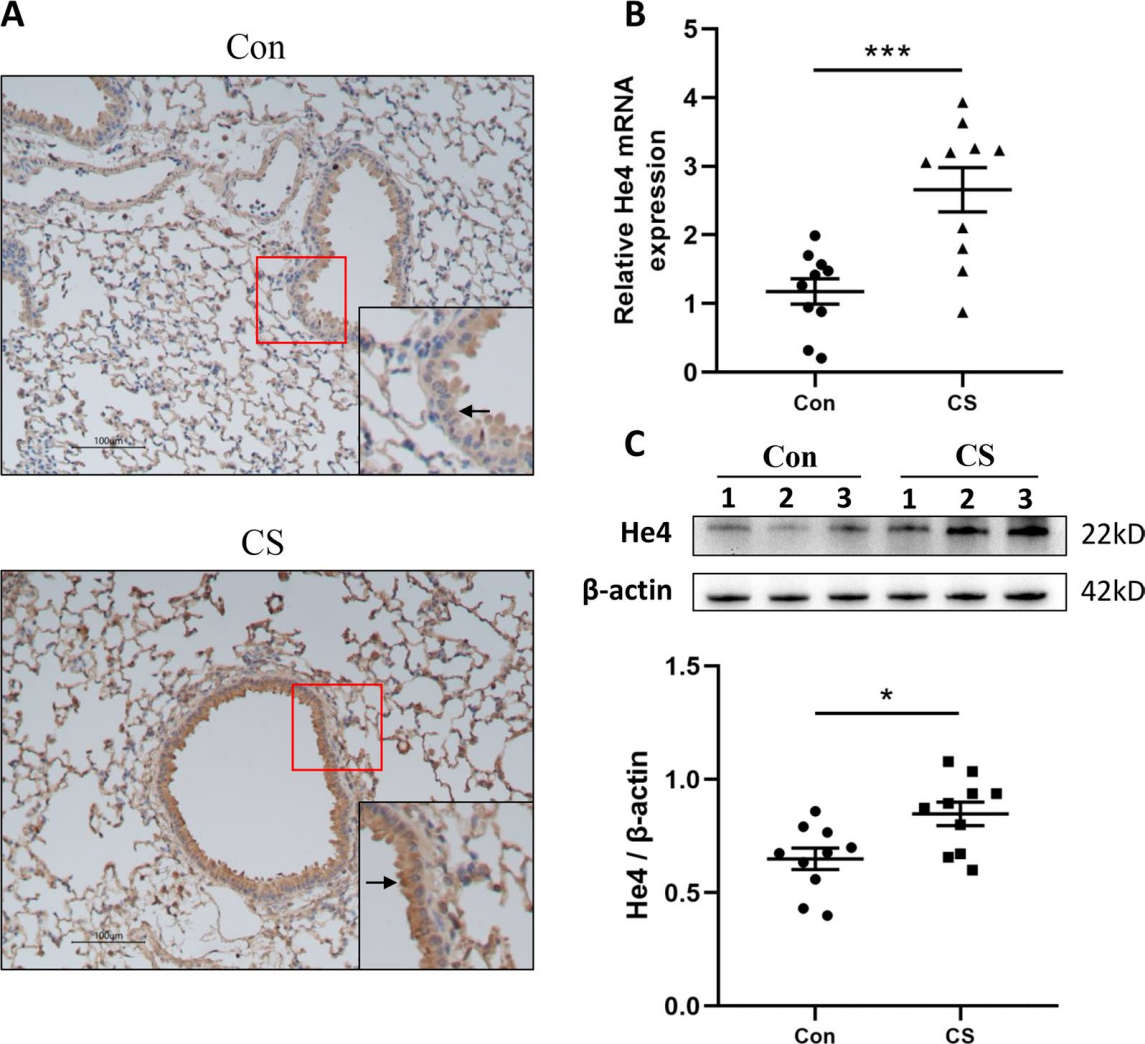
Enhanced He4 expression in CS-exposed mice. **A** Representative immunohistochemical analysis of He4 in the mouse lung sections are shown. Compared to controls, the intensity of tan staining indicating He4 expression was significantly increased in bronchial epithelium of CS-exposed mice. The staining alteration was clearly marked by the arrowheads. magnification = × 100. The red-boxed area indicates a region of higher magnification. **B** He4 mRNA expression was upregulated in the lung tissues of CS-exposed mice. **C** Representative image of Western blots against He4 was displayed and the band intensity was quantified using ImageJ. Each lane represents a different lung tissue subject. Data are presented as mean ± SEM, n = 10 for controls and n = 10 for CS-exposed mice. P-values were calculated using Student’s t-test. *P < 0.05 and ***P < 0.001 represent significant differences compared to controls. He4, human epididymis protein 4; Con, control mice; CS, cigarette smoke exposed mice

The establishment of the passive smoking mouse model has been confirmed by the detection of lung function and pathological examination [24]. We assessed the He4 expression in the lung tissues of 10 model mice and 10 control mice. The immunohistochemical analysis indicated more significant staining against He4 in the bronchial epithelium of cigarette smoke (CS)-exposed mice relative to controls (Fig. 2A). Furthermore, consistent with the results of immunohistochemical staining, the mRNA and protein expression of He4 were significantly enhanced in CS-exposed mice than air controls as validated by PCR and western blot (Fig. 2B, C).

### CSE enhanced HE4 expression and release in HBEs but not in HPFs

**Fig. 3:**
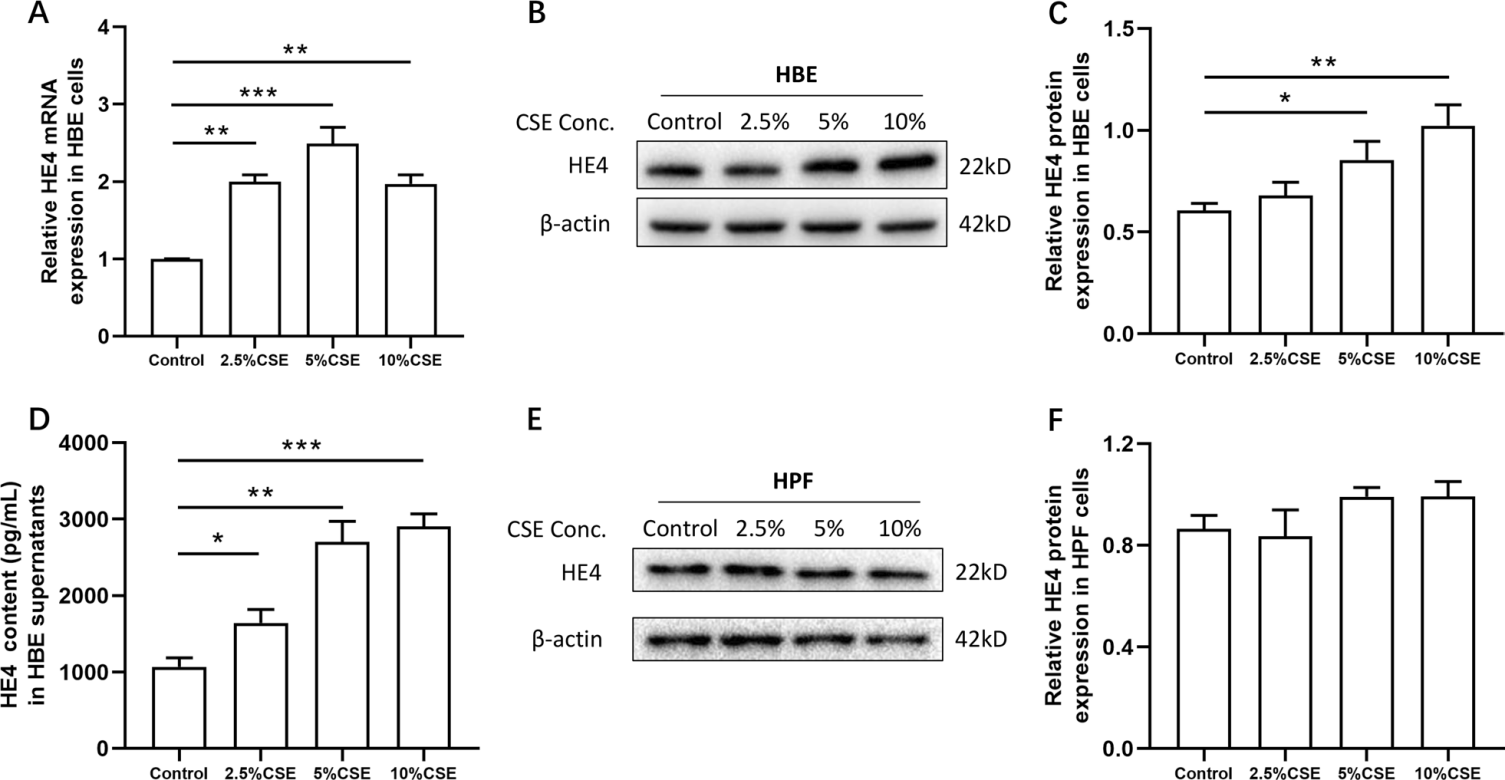
CSE enhanced HE4 expression in HBE cells but not in HPF cells. **A** Different concentrations of CSE increased HE4 mRNA expression in HBE cells (n = 3). **B, C** The image of western blot was shown and the band intensity was quantified by ImageJ (n = 3). Compared with controls, 5% CSE and 10% CSE markedly enhanced HE4 protein expression in HBE cells. **D** The secretory HE4 levels were detected in collected CSE-exposed HBE supernatants using ELISA (n = 4). **E, F** CSE had no obvious impact on HE4 expression in HPF cells (n = 3). Data are expressed as mean ± SEM. P-values were calculated using one-way ANOVA followed by Newman–Keuls test. *P < 0.05, **P < 0.01, and ***P < 0.001 represent significant differences relative to controls. CSE, cigarette smoke extract; HE4, human epididymis protein 4; HBE, human bronchial epithelial; HPF, human pulmonary fibroblast

Considering the main expression of HE4 in the bronchial epithelium, we investigated the effect of CSE on HBE cells and HPF cells. HBEs and HPFs were incubated with different concentrations of CSE (0, 2.5%, 5% and 10%) for 24 h. The cell viability was firstly detected by CCK-8 assay, which demonstrated that CSE concentration no more than 10% had no influence on cell viability (Additional file 1: Fig. S1A, B). And then the intracellular expression of HE4 was identified to be significantly elevated both at mRNA and protein levels by CSE treatment, especially at 5% CSE and 10% CSE (Fig. 3A–C). The secretory HE4 level in culture supernatant was also confirmed to be remarkably increased at different concentrations of CSE (Fig. 3D). Interestingly, CSE had no obvious influence on the HE4 expression in HPFs by the detection of western blot (Fig. 3E, F).

### Intervening HE4 expression affected IL-6 release via phosphorylation of NFκB-p65 in HBEs

**Fig. 4:**
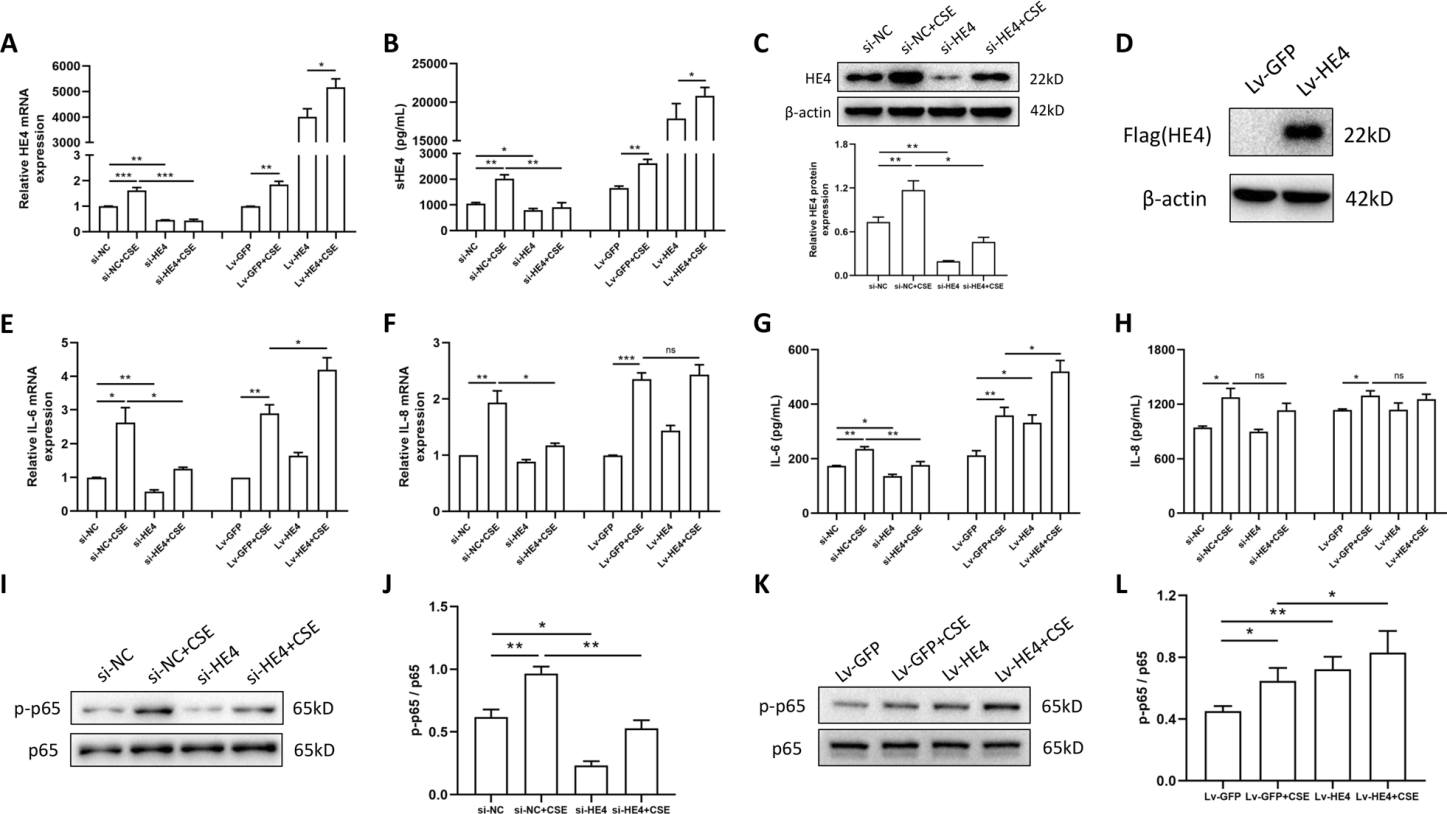
HE4 affected IL-6 release in HBE cells through phosphorylation of NFκB-p65. **A–C** The transfection of siRNA in HBE cells significantly lowered HE4 mRNA expression (n = 3), secretory level (n = 3) and intracellular protein expression (n = 4). **A, B, D** Infecting lentivirus expressing HE4 markedly enhanced HE4 mRNA expression (n = 3), secretory level (n = 4) and intracellular protein expression (n = 4). The western blot images were shown and quantified by ImageJ. **E, G** HE4 knockdown mitigated IL-6 expression while HE4 overexpression augmented IL-6 expression in HBE cells both in mRNA level (n = 3) and secretory protein level (n = 4). **F, H** Intervening HE4 expression had no significant impact on IL-8 expression, although reducing HE4 expression can alleviate CSE-induced IL-8 elevation at mRNA level (n = 3–4). **I, J** Knockdown of HE4 expression reduced phosphorylation of NFκB-p65 relative to the expression of p65 (n = 4). **K, L** Overexpression of HE4 increased phosphorylation of NFκB-p65 (n = 5). Data are expressed as mean ± SEM. P-values were calculated using one-way ANOVA followed by Newman–Keuls test. *P < 0.05, **P < 0.01, and ***P < 0.001 represent significant differences. HE4, human epididymis protein 4; si, small interfering RNA; NC, negative control; Lv-GFP, lentivirus with empty vector carrying green fluorescent protein; Lv-HE4, lentivirus expressing HE4; CSE, cigarette smoke extract; HBE, human bronchial epithelial; IL-6, interleukin-6; IL-8, interleukin-8

Here, we constructed transient-transfected HBE cells of HE4 knockdown and stable-infected HBE cells of HE4 overexpression through siRNA targeting HE4 and lentivirus overexpressing HE4 respectively. The intracellular HE4 mRNA and protein expressions were measured to be markedly decreased in siRNA-transfected HBEs (Fig. 4A, C), whereas significantly up-regulated in lentivirus-infected HBEs (Fig. 4A, D). The secretory levels of HE4 in the culture supernatants were determined to present the same changes as intracellular levels (Fig. 4B). After combining the treatments of 10% CSE for 24 h, the effects of siRNA and lentivirus were similarly remarkable.

The effects of HE4 knockdown on inflammatory cytokines in HBEs were investigated. As displayed in Fig. 4E and F, downregulating the HE4 expression could alleviate IL-6 mRNA level in HBEs regardless of CSE treatment, while only mitigated CSE-induced elevation for IL-8 mRNA. Furthermore, reduced HE4 expression alleviated IL-6 secretion in HBEs, but had no impact on IL-8 release (Fig. 4G, H). Meanwhile, the visibly attenuated phosphorylated levels of NFκB-p65 was observed in siRNA-HE4 treated HBEs with or without stimulation of CSE (Fig. 4I, J).

In experiments of HE4 overexpression by lentivirus, contrary to the knockdown experiments, upregulating HE4 facilitated CSE-induced IL-6 expression but not IL-8 at mRNA levels (Fig. 4E, F). Meanwhile, IL-6 release was enhanced by HE4 overexpression, whereas IL-8 secretion was not influenced (Fig. 4G, H). Moreover, overexpressing HE4 promoted phosphorylation of NFκB-p65 in HBEs and CSE-exposed HBEs (Fig. 4K, L). To further investigate the role of NFκB-p65 in HE4-regulated IL-6 increase, we administrated HBEs with Bay 11-7821, a selective inhibitor of NFκB pathway. Results indicated that different concentrations of Bay 11-7821 could reduce the phosphorylated level of NFκB-p65 (Additional file 2: Fig. S2A, B), and that treatment with Bay 11-7821 at 10 μM could significantly alleviate IL-6 secretion in HE4-lentivirus infected HBE cells (Additional file 2: Fig. S2C).

Collectively, two-way intervention experiments on HE4 expression in HBEs confirmed the positive regulation of HE4 on IL-6 expression and phosphorylation of NFκB-p65. Therefore, we speculated that HE4 promoted IL-6 release in HBEs potentially via phosphorylation of NFκB-p65.

### Secretory HE4 promoted fibroblast differentiation and proliferation

**Fig. 5:**
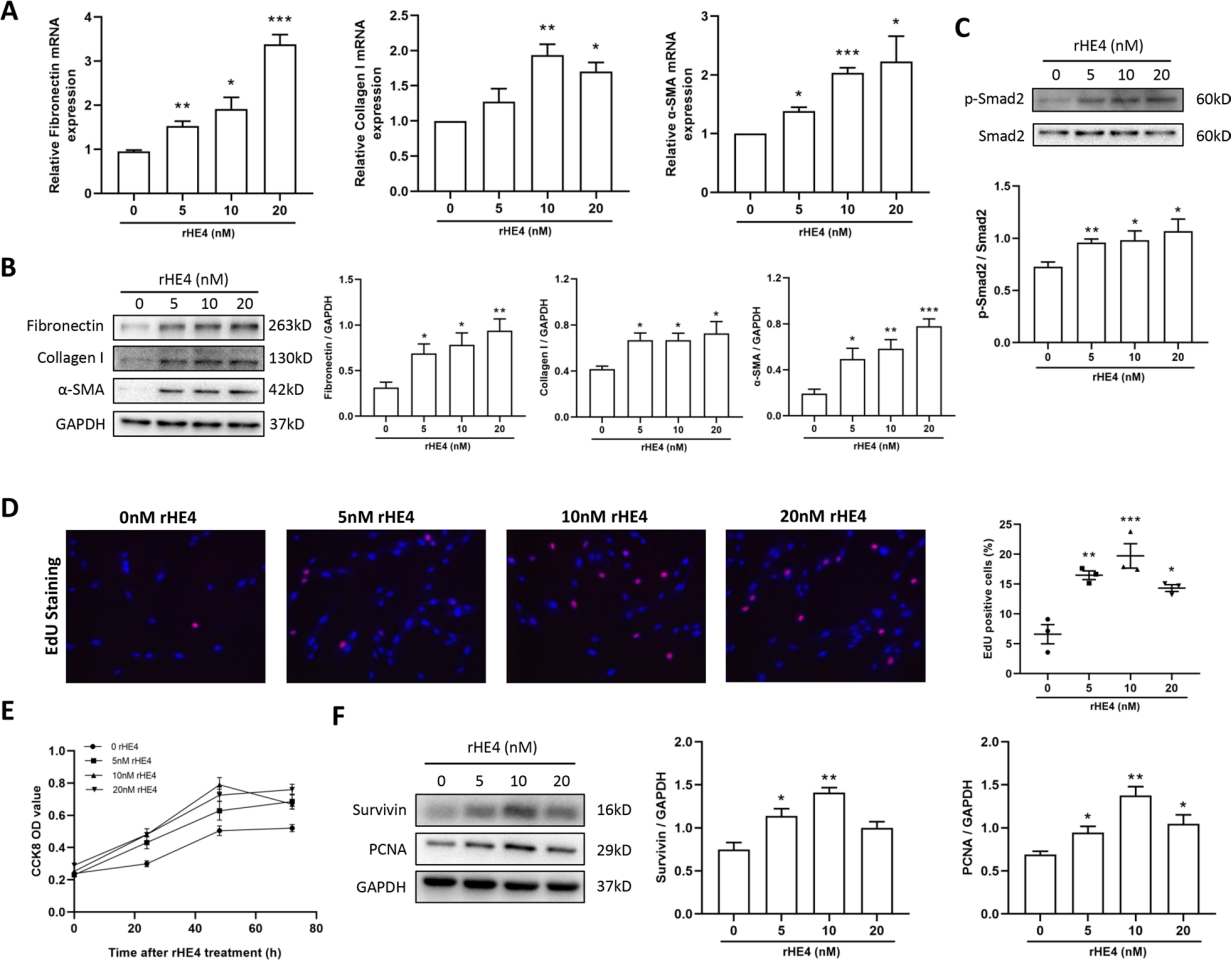
Recombinant HE4 promoted fibroblast-myofibroblast transition and fibroblastic proliferation. **A, B** Different concentrations of rHE4 facilitated fibronectin, collagen I and α-SMA expression both in mRNA level (n = 3) and protein level (n = 4). The representative western blot images were shown and the band intensity was quantified by ImageJ. **C** Treatment with rHE4 in HPF cells enhanced phosphorylation of Smad2 (n = 4). **D** Typical images of EdU-staining HPFs followed by rHE4 treatment for 48 h and the quantitative graph were shown (n = 3). magnification = × 200. **E** CCK8 proliferation curve signified the pro-proliferative effect of rHE4 (n = 4). HPF was stimulated by rHE4 for 0 h, 24 h, 48 h and 72 h. **F** Representative western blot images of PCNA and Survivin were displayed and quantified by ImageJ (n = 3). Data are presented as mean ± SEM. P-values were calculated using one-way ANOVA followed by Newman–Keuls test. *P < 0.05, **P < 0.01, and ***P < 0.001 represent significant differences. rHE4, recombinant human epididymis protein 4; EdU, 5-ethynyl-2′-deoxyuridine; CCK8, Cell Counting Kit-8; PCNA, proliferating cell nuclear antigen; HPF, human pulmonary fibroblast

To explore whether secretory HE4 (sHE4) released from CSE-induced HBEs affects fibroblast function, we treated HPFs with rHE4 in vitro to investigate the role of sHE4 on fibroblast-myofibroblast transition (FMT) and fibroblastic proliferation, which have been reported to facilitate airway remodeling [27–29]. First, we examined the expression of typical activation markers of FMT through treating HPFs with different concentrations of rHE4 (0, 5 nM, 10 nM, 20 nM) for 48 h. Interestingly, the results showed that compared with the controls, administration of rHE4 at any concentration substantially enhanced the expressions of fibronectin, collagen I and α-SMA, as indicated by RT-qPCR (Fig. 5A) and western blot analysis (Fig. 5B). To further understand the mechanism by which rHE4 promotes FMT, we detected the phosphorylated level of Smad2, which is considered to be the most influential signaling molecule affecting fibroblast differentiation. As expected, different concentration of rHE4 markedly increased phosphorylation of Smad2 (Fig. 5C). Therefore, the data above supported the hypothesis that sHE4 promoted the FMT by elevating the phosphorylated level of Smad2.

In addition, we conducted an EdU staining assay to detect the proliferative ability of HPFs treated by rHE4 for 48 h. The results demonstrated that relative to control treatment, the proliferative cells with EdU-positive staining were prominently increased at various concentration of rHE4, especially at 10 nM rHE4 (Fig. 5D). Meanwhile, CCK8 proliferation curve also confirmed the pro-proliferative effect of rHE4 in HPFs at different time point (Fig. 5E). To further verify the result, we assessed representative marker molecules of cell proliferation using western blot. Parallel to previous result, stimulating HPFs with different concentration of rHE4 could enhance the expressions of Survivin and PCNA at protein level (Fig. 5F).

### CSE-induced HE4 increase in HBEs was mediated by oxidative stress

**Fig. 6:**
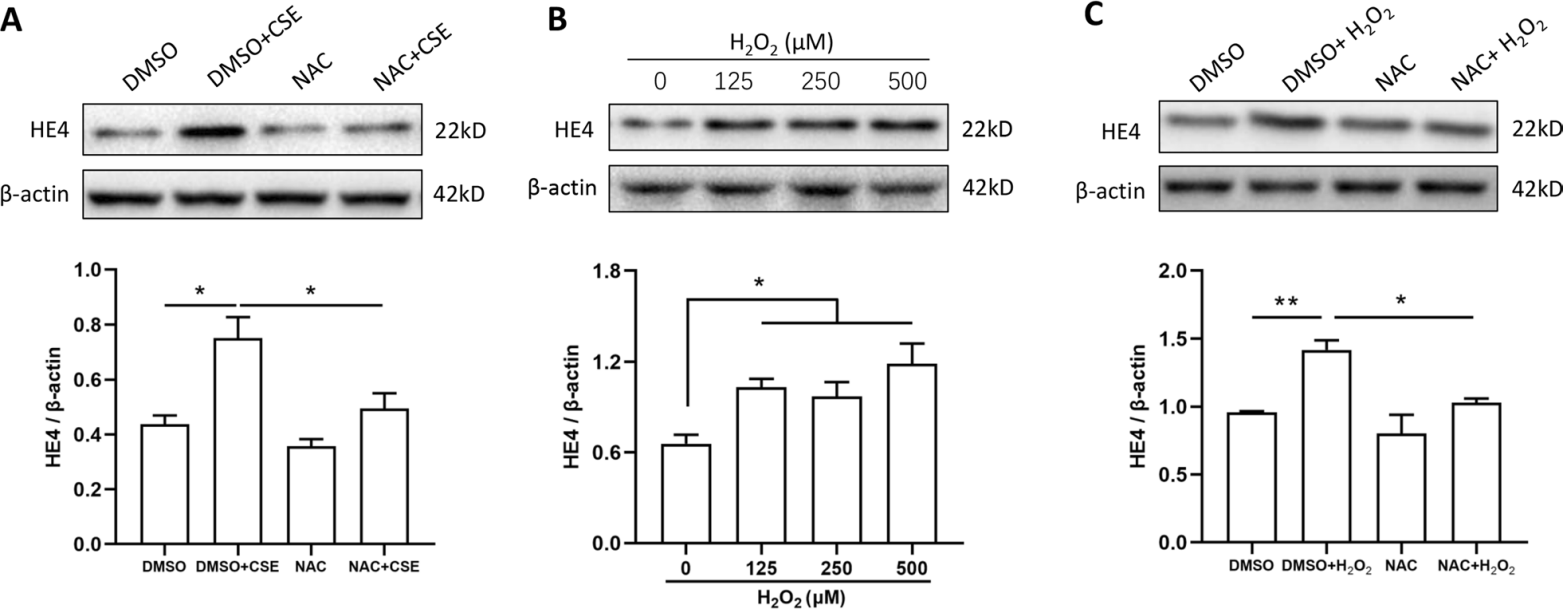
Elevated HE4 expression in CSE-exposed HBE cells was mediated by oxidative stress. **A** Pretreatment of NAC overtly alleviated CSE-induced HE4 increase (n = 3). **B** Different concentrations of H_2_O_2_ promoted HE4 expression (n = 3). **C** Pretreating NAC significantly mitigated H_2_O_2_-induced HE4 up-regulation (n = 3). Data are expressed as mean ± SEM. P-values were calculated using one-way ANOVA followed by Newman–Keuls test. *P < 0.05 and **P < 0.01 represent significant differences. HE4, human epididymis protein 4; CSE, cigarette smoke extract; HBE, human bronchial epithelial; DMSO, dimethyl sulfoxide; NAC, N-acetylcysteine

To study the potential mechanism involved in the elevated HE4 expression by cigarette smoke in bronchial epithelial cells, HBEs were cultured with CSE as an in vitro model. Given that oxidative stress is one of the important mechanisms for the impairment of epithelium cell function by cigarette smoke, we administrated 10% CSE for 24 h and NAC, a ROS scavenger, in HBEs to observe the HE4 expression. Result indicated that pretreatment with 3 mM NAC in HBEs could remarkably alleviate the CSE-induced increase in HE4 expression (Fig. 6A). Then, HBEs were stimulated with different concentrations of H_2_O_2_ for 24 h, which has been regarded as a typical ROS generator. Cell viability was not affected by H_2_O_2_ indicated by CCK-8 assay (Additional file 1: Fig. S1C). The marked increase in HE4 expression was observed at various concentration of H_2_O_2_ (Fig. 6B), whereas 3 mM NAC could likewise mitigate the increase in HE4 expression induced by 500 μM H_2_O_2_ (Fig. 6C). Collectively, these results strongly suggested that cigarette smoke promoted HE4 expression in bronchial epithelial cells, which was mediated by oxidative stress.

## Discussion

**Fig. 7:**
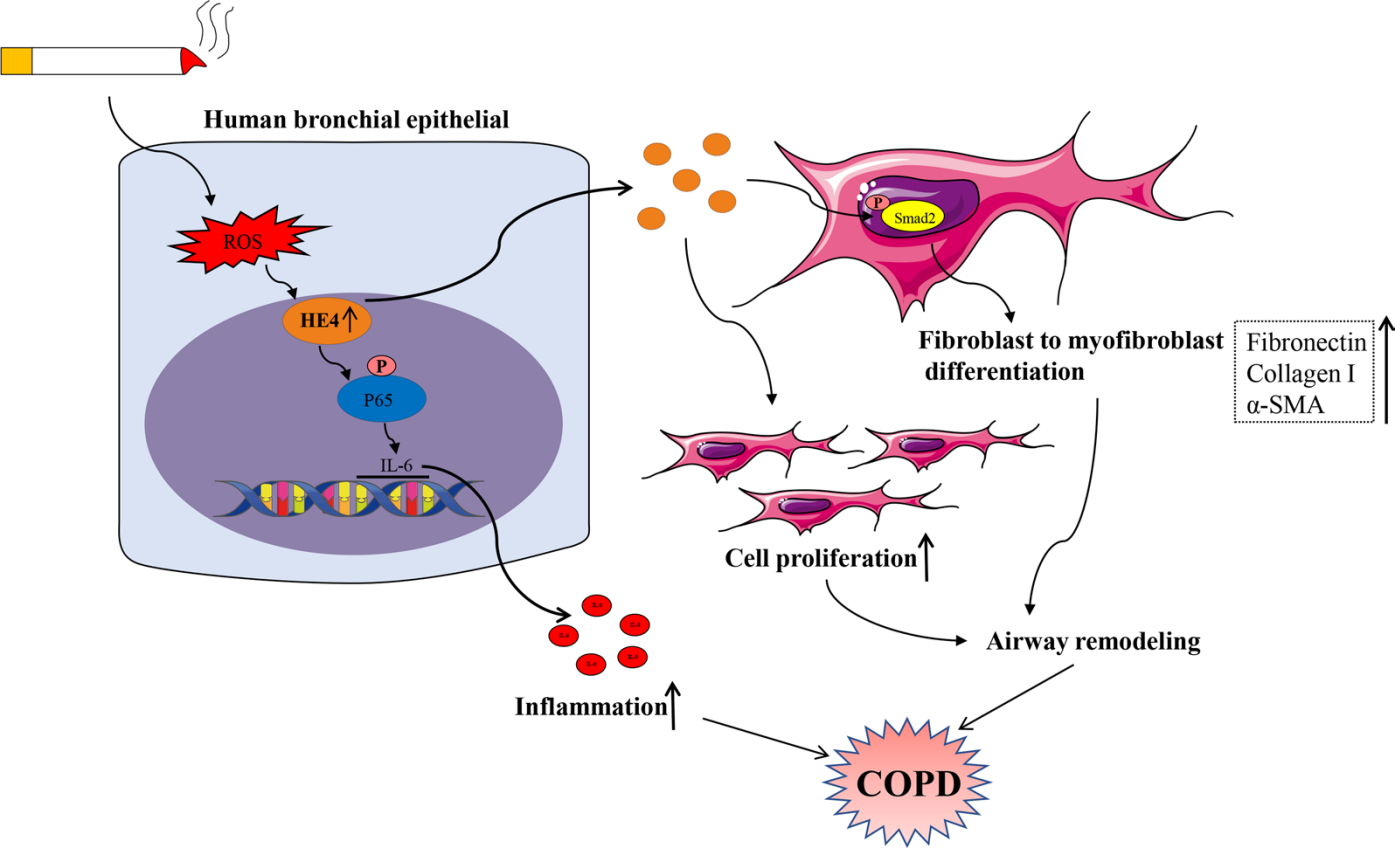
The diagram of mechanism underlying HE4 regulation in COPD. Cigarette smoke promotes HE4 expression and secretion in bronchial epithelial cells partly through oxidative stress, and then mediated IL-6 production via the phosphorylation of NFκB-p65 to participate in airway inflammation. The elevated HE4 release from bronchial epithelial cells facilitates fibroblast to myofibroblast differentiation and fibroblastic proliferation to aggravate airway remodeling. HE4, human epididymis protein 4; ROS, reactive oxygen species; IL-6, interleukin-6; COPD, chronic obstructive pulmonary disease

In the current study, we first found that the HE4 expression and secretion were increased in the bronchial epithelium and peripheral blood plasma of COPD patients, and meanwhile discovered the plasma HE4 was negatively associated with lung function. Moreover, the same increase of HE4 expression was observed in CS-exposed mice and CSE-treated HBE cells, compared with controls. In in vitro experiments, we confirmed that cigarette smoke promoted HE4 expression and secretion in bronchial epithelial cells, which was partly mediated by oxidative stress, and then facilitated IL-6 production via elevating phosphorylated level of NFκB-p65 to participate in airway inflammation. The increased secretory HE4 derived from bronchial epithelial cells prompted fibroblast to myofibroblast differentiation and fibroblastic proliferation to act in airway remodeling (Fig. 7). These findings indicated the fundamental role of HE4 in airway inflammation and remodeling associated with COPD.

COPD is one of the most noticeable public health challenges, with high prevalence, disability and mortality, and cigarette smoke remains the predominantly attributable risk factor [3]. Previous studies have reported that higher ROS content was discovered in the lung tissue of smokers with or without COPD than healthy controls [30], and CSE could provoke ROS production, which may further promote inflammatory gene expression through activating oxidative stress-related signaling pathway [31]. Here, we verified that pretreatment with NAC, a ROS scavenger, could alleviate the increased HE4 expression in CSE-exposed or H_2_O_2_-treated HBE cells, which suggested the regulation role of oxidative stress in HE4 expression. More, Bordin et al. found that in erythrocytes from endometriosis patients, the HE4 level was inversely correlated with glutathione level [32], an important antioxidant in the body, likewise indicating the possible association between HE4 and oxidative stress.

Chronic inflammation, as a hallmark feature for COPD, predominantly affecting the lung parenchyma and peripheral airways, can take place in various types of cell involving both immune and structural cells, like alveolar macrophages and bronchial epithelial cells [14, 33]. In response to chronic CS exposure, the bronchial epithelial cells serving as the first defense barrier are activated and secret a mass of proinflammatory mediators including IL-6 and IL-8 [13]. Previous studies have shown that IL-6 and IL-8 were increased in COPD patients, and presented strong associations with airway obstruction and lung function [34–36]. In our research, we confirmed that HE4 expression was up-regulated in the lung tissue and peripheral blood plasma of COPD patients relative to healthy controls, and plasma HE4 presented distinct negative association with lung function, indirectly suggesting the possible role of HE4 in COPD pathogenesis and inflammation. Meanwhile, we substantiated that HE4 promoted IL-6 release in in vitro experiments. HE4 was reported to positively regulate inflammatory cytokines including IL-6 in cystic fibrosis [37], which supported our result to some extent. Furthermore, exposure of bronchial epithelium to CS results in the activation of numerous redox-associated intracellular signaling pathways including mitogen-activated protein kinases (MAPK) and nuclear factor kappa-light-chain-enhancer of activated B cells (NFκB), which thereafter trigger inflammation [38]. Here, we displayed that HE4-regulated IL-6 increase was mediated by phosphorylation of NFκB-p65, which was consistent with previous studies [20, 39].

Remodeled airways in COPD are characterized by an alteration in the epithelial injury, goblet cell hyperplasia, airway smooth muscle proliferation, activation and proliferation of myofibroblast, collagen deposition and reticular basement membrane thickening [40, 41]. Airway remodeling contributes to progressive and irreversible airflow limitation [42]. During this process, fibroblasts originating from the respiratory tracts contribute to the formation of airway fibrosis, manifesting as the promotions of myo-fibroblast differentiation, collagen release, and cell proliferation [16, 43]. The fibroblast-to-myofibroblast transition (FMT) was reported to stimulate the epithelium to release more inflammatory factors [44], and meanwhile the cytokines like IL-6 released from epithelium in return promote the fibroblastic differentiation and collagen deposition [16], which may form a vicious cycle between airway inflammation and remodeling. In the current study, we verified that CSE-induced HE4 increase enhanced IL-6 expression and release in bronchial epithelial cells, which can be reasonably speculated to further promote the fibroblastic differentiation. In bronchial epithelial cells, CSE simultaneously facilitated the secretion of HE4 to the outside of cells besides the release of IL-6. Whether the secreted HE4 induces FMT was investigated in our study.

HE4 has been deemed as a crucial fibrosis-associated molecule, participating in various fibrotic disorders, and was expected to be a promising therapeutic target. During the cardiac fibrosis in dilated cardiomyopathy, HE4 functions as a secretory factor, activating cardiac fibroblasts, thereby inducing cardiac interstitial fibrosis [25]. In kidney fibrosis, HE4 was confirmed to inhibit the capacity to degrade type I collagen by targeting two serine proteases, Prss35 and Prss23 [45]. Moreover, in patients with cystic fibrosis, the concentration of serum HE4 was positively correlated with overall disease severity [21]. However, to date, there presents minor evidence indicating the role of HE4 in fibrosis-associated respiratory diseases. Although Raghu et al. reported the moderate correlation between HE4 and diffusing capacity of the lung for carbon monoxide in patients with idiopathic pulmonary fibrosis [46], many questions about what cell HE4 exerts the specific function in and what mechanism HE4 acts by remained unidentified. Here, we demonstrated that the secretory HE4 played a critical role in promoting FMT and collagen deposition, as indicated by the enhanced expression in α-SMA, collagen I and fibronectin via increasing the phosphorylated level of Smad2. In addition, we unexpectedly found the pro-proliferative role of sHE4 in fibroblasts. Accordingly, the CS-induced sHE4 secreted from bronchial epithelial cells may aggravate airway remodeling in COPD through directly facilitating fibroblastic differentiation and proliferation, as well as collagen deposition.

There remain some limitations in this study. First of all, we verified the expression level of HE4 in the lung tissue in CS-exposed mice, while the mechanism investigations in in vivo experiments haven’t been conducted using genetic intervention mice targeting HE4. In addition, although we eliminated the gender interference in HE4 expression levels between COPD patients or CS-exposed mice and controls by ensuring the ratio of male to female with no difference, whether different genders have an effect on the expression of HE4 in the airway of COPD patients and whether it is related to the level of estrogen remains to be investigated. Furthermore, to explore the effect of sHE4 secreted by HBE on fibroblast, it is ideal to collect the supernatant of CSE-treated HBEs and culture with HPF medium in a certain ratio. But due to the lack of HE4 neutralizing antibodies, there is no way to determine whether the changes of fibroblastic function are subjected to sHE4. Therefore, we here used recombinant HE4 protein instead to explore the function of sHE4 on HPF and it’s unknown whether the findings of rHE4 can be extrapolated to sHE4. Finally, the specific receptor in fibroblast to which sHE4 directly binds requires further research in the future.

## Conclusion

We highlighted the role of HE4 in COPD pathogenesis, and demonstrated that HE4 promoted airway inflammation and remodeling through facilitating the release of inflammatory cytokine IL-6 in bronchial epithelial cells and myo-differentiation and proliferation in fibroblasts. These results indicated the potentially diagnostic and therapeutic value of HE4 in COPD.

## Supplementary Information

**Additional file 1. Figure S1. **Cell viability in HBE or HPF cells under different treatments. A, B Cell viability was not affected both in HBE cells (n = 4) and HPF cells (n = 4) under different concentrations of CSE for 24 h. C Treatment with various concentrations of H_2_O_2_ for 24 h conducted no obvious influence on HBE cell viability (n = 4). Data are expressed as mean ± SEM. P-values were calculated using one-way ANOVA followed by Newman–Keuls test. CSE, cigarette smoke extract; HBE, human bronchial epithelial; HPF, human pulmonary fibroblast.

**Additional file 2. Figure S2. **NFκB inhibitor alleviated IL-6 elevation in the supernatant of HBE cells infected by lentivirus of HE4. A, B The phosphorylation of NFκB-p65 was mitigated in HBE cells treated with different concentrations of Bay 11-7821, a NFκB inhibitor. Representative western blot images were shown and the band was quantified using ImageJ (n = 3). C ELISA assay has demonstrated that treatment with Bay 11-7821 at 10 μM markedly reduced IL-6 level in the supernatant of HE4-lentivirus infected HBE cells (n = 4). Data are expressed as mean ± SEM. P-values were calculated using one-way ANOVA followed by Newman–Keuls test. *P < 0.05, **P < 0.01, and ***P < 0.001 represent significant differences. HE4, human epididymis protein 4; HBE, human bronchial epithelial; IL-6, interleukin-6; Bay, Bay 11-7821; DMSO, dimethyl sulfoxide.

## Data Availability

The datasets used and/or analysed during the current study are available from the corresponding author on reasonable request.

## Abbreviations

COPD: Chronic obstructive pulmonary disease
CSE: Cigarette smoke extract
EdU: 5-Ethynyl-2′-deoxyuridine
ELISA: Enzyme-linked immunosorbent assay
FEV_1_: Forced expiratory volume in 1 s
FMT: Fibroblast-myofibroblast transition
HE4: Human epididymis protein 4
IL-6: Interleukin-6
IL-8: Interleukin-8
ROS: Reactive oxygen species
