# Supplementary figures and images for: Human epididymis protein 4 aggravates airway inflammation and remodeling in chronic obstructive pulmonary disease
Source: Respir Res. 2022 May 12;23:120. doi: 10.1186/s12931-022-02040-7 (PMC9097053; doi:10.1186/s12931-022-02040-7)

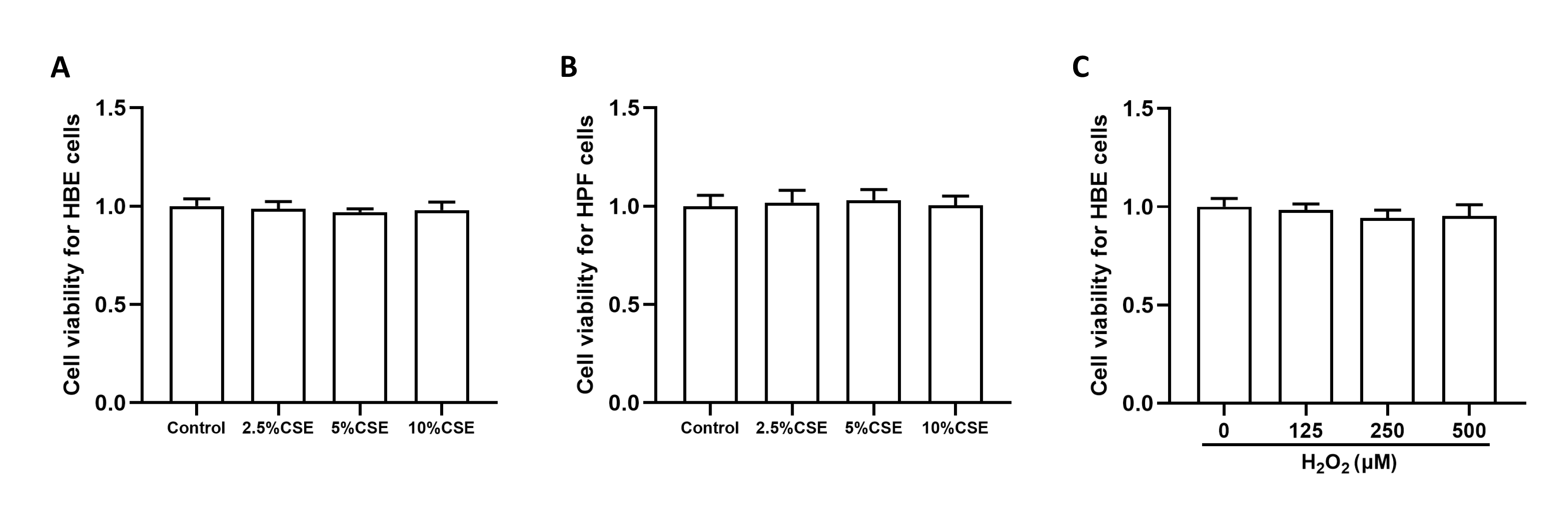

Supplement: Supplementary file 1 — Additional file 1. Figure S1. Cell viability in HBE or HPF cells under different treatments. A, B Cell viability was not affected both in HBE cells (n = 4) and HPF cells (n = 4) under different concentrations of CSE for 24 h. C Treatment with various concentrations of H2O2 for 24 h conducted no obvious influence on HBE cell viability (n = 4). Data are expressed as mean ± SEM. P-values were calculated using one-way ANOVA followed by Newman–Keuls test. CSE, cigarette smoke extract; HBE, human bronchial epithelial; HPF, human pulmonary fibroblast. [file 12931_2022_2040_MOESM1_ESM.tif]

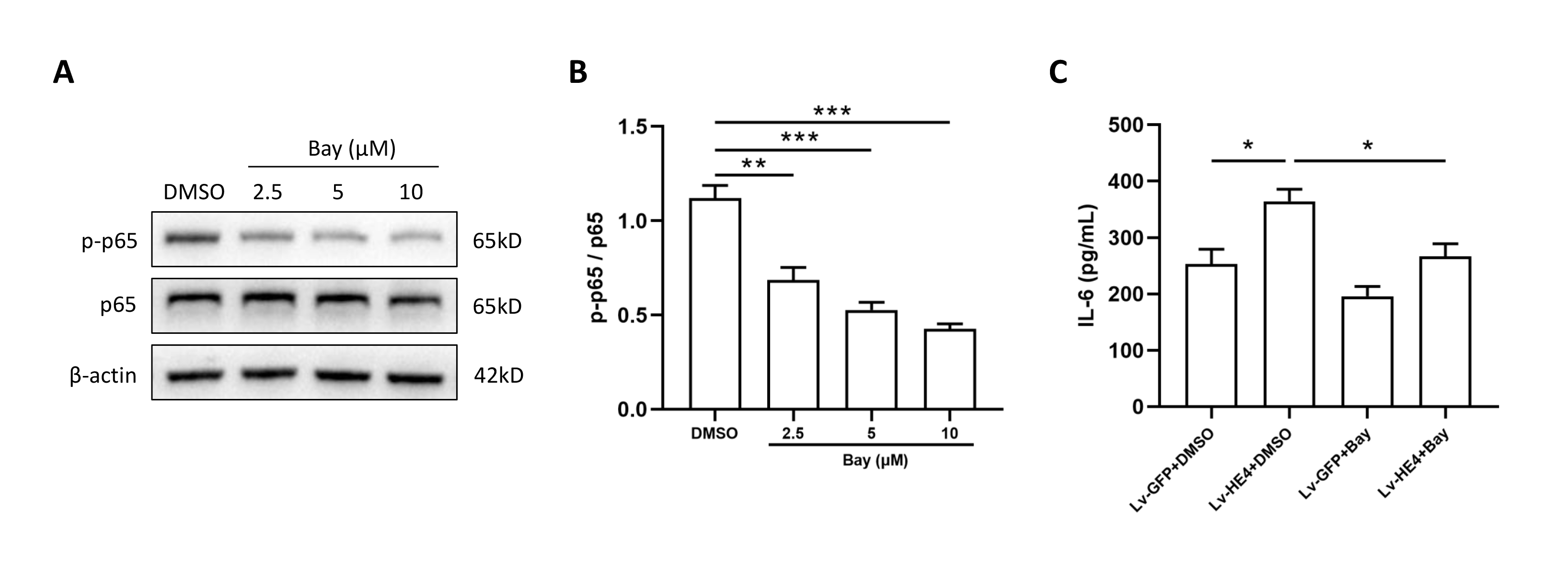

Supplement: Supplementary file 2 — Additional file 2. Figure S2. NFκB inhibitor alleviated IL-6 elevation in the supernatant of HBE cells infected by lentivirus of HE4. A, B The phosphorylation of NFκB-p65 was mitigated in HBE cells treated with different concentrations of Bay 11-7821, a NFκB inhibitor. Representative western blot images were shown and the band was quantified using ImageJ (n = 3). C ELISA assay has demonstrated that treatment with Bay 11-7821 at 10 μM markedly reduced IL-6 level in the supernatant of HE4-lentivirus infected HBE cells (n = 4). Data are expressed as mean ± SEM. P-values were calculated using one-way ANOVA followed by Newman–Keuls test. *P < 0.05, **P < 0.01, and ***P < 0.001 represent significant differences. HE4, human epididymis protein 4; HBE, human bronchial epithelial; IL-6, interleukin-6; Bay, Bay 11-7821; DMSO, dimethyl sulfoxide. [file 12931_2022_2040_MOESM2_ESM.tif]
